# Are Health-Related Tweets Evidence Based? Review and Analysis of Health-Related Tweets on Twitter

**DOI:** 10.2196/jmir.4898

**Published:** 2015-10-29

**Authors:** Khalid A Alnemer, Waleed M Alhuzaim, Ahmed A Alnemer, Bader B Alharbi, Abdulrahman S Bawazir, Omar R Barayyan, Faisal K Balaraj

**Affiliations:** ^1^Al-Imam Mohammad Ibn Saud Islamic UniversityRiyadhSaudi Arabia; ^2^Department of Internal MedicineAl-Imam Mohammad Ibn Saud Islamic UniversityRiyadhSaudi Arabia; ^3^Kind Saud UniversityRiyadhSaudi Arabia

**Keywords:** health, diseases, daily medical information, medical accounts, health accounts, doctor accounts, nutrition accounts

## Abstract

**Background:**

Health care professionals are utilizing Twitter to communicate, develop disease surveillance systems, and mine health-related information. The immediate users of this health information is the general public, including patients. This necessitates the validation of health-related tweets by health care professionals to ensure they are evidence based and to avoid the use of noncredible information as a basis for critical decisions.

**Objective:**

The aim of this study was to evaluate health-related tweets on Twitter for validity (evidence based) and to create awareness in the community regarding the importance of evidence-based health-related tweets.

**Methods:**

All tweets containing health-related information in the Arabic language posted April 1-5, 2015, were mined from Twitter. The tweets were classified based on popularity, activity, interaction, and frequency to obtain 25 Twitter accounts (8 physician accounts, 10 nonofficial health institute accounts, 4 dietitian accounts, and 3 government institute accounts) and 625 tweets. These tweets were evaluated by 3 American Board–certified medical consultants and a score was generated (true/false) and interobserver agreement was calculated.

**Results:**

A total of 625 health-related Arabic-language tweets were identified from 8 physician accounts, 10 nonofficial health institute accounts, 4 dietician accounts, and 3 government institute accounts. The reviewers labeled 320 (51.2%) tweets as false and 305 (48.8%) tweets as true. Comparative analysis of tweets by account type showed 60 of 75 (80%) tweets by government institutes, 124 of 201 (61.7%) tweets by physicians, and 42 of 101 (41.6%) tweets by dieticians were true. The interobserver agreement was moderate (range 0.78-0.22). More than half of the health-related tweets (169/248, 68.1%) from nonofficial health institutes and dietician accounts (59/101, 58.4%) were false. Tweets by the physicians were more likely to be rated “true” compared to other groups (*P*<.001).

**Conclusions:**

Approximately half of the medical tweets from professional accounts on Twitter were found to be false based on expert review. Furthermore, most of the evidence-based health-related tweets are posted by government institutes and physicians.

## Introduction

Twitter it is a free social networking website established in July 2006, which enables users to write and read online posts (known as “tweets”) that are limited to 140 characters. Tweets can be posted via the Web, instant online message, or mobile phone. Twitter has more than 500 million active users who generate more than 340 million tweets and 1.6 billion search queries per day.

Several health care professionals in the Middle Eastern countries use social media, especially Twitter, because of its ability to connect seamlessly with colleagues, patients, and other medical professionals. It is also a great resource to educate the public, track disease outbreaks, collect real-time health data, recruit study participants, recognize misuse of antibiotics, and gain more knowledge on health-related topics.

Several studies have examined the content of health-related tweets on Twitter. A study that investigated all posts with the words “Ebola” and “prevention” or “cure” from Guinea, Liberia, and Nigeria showed that the most common misinformation was that Ebola might be cured by the plant ewedu or by blood transfusion [1]. A study in 2010 investigated status updated from 52,153 tweets with the combination “flu + antibiotics” and “cold + antibiotics” associated with misinformation. Results showed a total of 172,571 and 850,375 followers of misinformation, respectively, for the 2 combinations [2].

Tweets were primarily used to disseminate information from credible sources, but were also a source of opinions and experiences [3]. A study conducted in Norway regarding the content and seriousness of tweets on chlamydia and HIV showed that 9 of 10 tweets on HIV were of serious nature and many of the tweets that were retweeted were facts [4]. A study conducted to evaluate opinions and knowledge regarding computed tomography radiation risk from 621 tweets posted by 557 accounts (doctors: 16%; health institute: 5%; patients: 6%; technologists: 1%; other users: 71%) showed that most tweets were not peer-reviewed, were posted by nonphysicians, and content was unfavorable [5].

A study conducted in 2014 at The John Hopkins University to analyze the content of 665 tweets on Twitter showed that 346 were health-related tweets, 53.2% were testable claims, 41.0% were news, 26.9% were commercial product or service, 17.6% were personal experience, and 17.1% were about wellness [6].

These studies indicate that the validity of health-related tweets on Twitter needs to be assessed, especially to check if the content represents a claim supported by evidence, a personal opinion, or other information. Thus, this study reports the results of a content analysis of health-related tweets on Twitter in Arabic.

## Methods

Twitter was chosen to investigate health-related tweets because it is the most common social media in the Gulf countries. Only tweets in Arabic were included in this study.

A manual approach was used to identify and categorize health-related tweets posted by health-related accounts, associated with either an organization or an individual user. These tasks were crucial for the identification, data collection, and categorization process for this study.

### Identification of Relevant Twitter Accounts

The relevant accounts were identified via a 4-step process. The first step involved a search of the Twitter website using the following search terms in Arabic: health, your health, agility, regimen, healthy diet, drugs , disease, diseases, drug, treatment, prohibited drugs, epidemic, inflammations, infection, medical information, doctors, hospitals, daily medical information, nutrition, medical accounts, health accounts, doctor accounts, and nutrition accounts (see Multimedia Appendix 1 for Arabic search terms). This search resulted in 203 tweets that were reviewed; those accounts whose identity could not be ascertained were excluded.

The second step involved selection of accounts based on:

1. Number of followers (minimum number was set to 250,000);
2. Activity (tweeted for the period of April 2015);
3. Interaction with other users; and
4. Frequency of tweets (health-related tweets on a daily basis).

This resulted in a list of 86 Twitter accounts: 31 physician accounts, 39 nonofficial health institute accounts, 6 dietitian accounts, 2 media accounts, and 8 government institute accounts.

The third step involved further examination of the 86 accounts by the following 3 criteria:

1. Popularity (most viewed);
2. Interaction with other users; and
3. Number of followers (minimum number was set to 45,000).

Accounts with a minimum of 45,000 followers were reviewed for 1 week to select those posting at least 5 health-related tweets per day with at least 100 retweets a week. This resulted in a list of 25 Twitter accounts: 8 physician accounts, 10 nonofficial health institute accounts, 4 dietitian accounts, and 3 government institute accounts.

#### Physician Twitter Accounts

Twitter accounts were identified as those whose description provided a Web link to their corresponding clinics/hospital website. In total, there were 8 physician Twitter accounts.

#### Nonofficial Health Institute Twitter Accounts

Twitter accounts that were identified as those whose description provided a Web link to a nonofficial health institute. These accounts had a range of 70,000 to 300,000 followers. There were a total of 10 such accounts.

#### Dietician Twitter Accounts

Twitter accounts that were identified as those whose description provided a Web link to their clinic/hospital website. These accounts had a range of 45,000 to 210,000 followers. There were a total of 4 such accounts.

#### Government Institute Twitter Accounts

Twitter accounts that were identified as those whose description provided a Web link to their corresponding governmental site (ie, ending with gov.sa). These accounts had a range of 43,000 to 843,000 followers. There were a total of 3 such accounts.

The final step involved selection of the first 5 health-related tweets daily for 5 days (April 1-5, 2015) from each of these 25 accounts. This resulted in a total of 625 tweets, which were integrated in a Microsoft Word file.

### Examination of Individual Tweets

The Word file was evaluated by 3 independent reviewers (American Board–certified consultants with more than 10 years of experience in medical practice collaborating with other specialty consultants in different fields, if needed) who were blind to the identity of the Twitter users during content analysis. The reviewers evaluated and labeled these tweets as false, true with weak evidence (ie, expert opinion), true with moderate evidence (ie, small randomized controlled trial [RCT], nonrandomized observational study, registry), or true with strong evidence (ie, many large RCTs).

This was followed by scoring of the tweets, a system that used the majority of the reviewer’s opinions to generate a score for each tweet. For example, if 2 of 3 experts decided on moderate evidence, moderate evidence was chosen as the score for the tweet.

If there was no majority in the reviewers’ opinion, the lower evidence level was chosen as the score. For example, if the 3 reviewers chose weak evidence, moderate evidence, and false, respectively, because the majority had ranked it as true, weak evidence was chosen as the score for the tweet.

Descriptive statistics were used to tabulate types of account and response of each reviewer. Comparative analysis of type of Twitter account and chi-square tests were used to determine statistical significance of the result. Interobserver agreement for the 3 independent reviewers was based on the following formula: (true/[true+false]).

## Results

**Figure 1:**
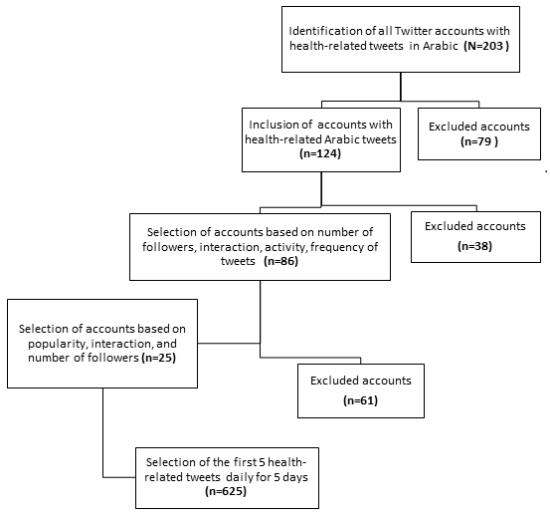
Collection of data flowchart.

The data collection process for this study is presented in Figure 1. Overall, 625 Arabic-language health-related tweets contributed by 25 user accounts were analyzed as defined in Table 1.

**Table 1:** Tweets by type of account (total tweets: N=625).

Account	n (%)
Physician	201 (32.2)
Government institute	75 (12.0)
Nonofficial health institute	248 (39.7)
Dietician	101 (16.2)

The evaluation of each health-related tweet and categorization into 1 of 4 categories (false, weak, moderate, or strong) by the 3 independent reviewers. In the absence of a majority within the true category, weak evidence was chosen as the score for the tweet (Table 2).

**Table 2:** Coding of tweets by reviewer.

Reviewers decision	False, n (%)	True, n (%)			Interobserver agreement
		Weak	Moderate	Strong	
Expert 1	268 (42.9)	332 (53.1)	20 (3.2)	5 (0.8)	0.57
Expert 2	140 (22.4)	226 (36.2)	173 (27.7)	86 (13.8)	0.78
Expert 3	488 (78.1)	62 (9.9)	72 (11.5)	3 (0.5)	0.22
Final decision	320 (51.2)	261 (41.8)	39 (6.2)	5 (0.8)	

More than half of the tweets (320/625, 51.2%) in this sample were not supported by medical evidence (Table 2). The interobserver agreement between the 3 independent reviewers ranged from 0.78 to 0.22 (Table 2).

Comparative analysis of tweets by account type showed that 60 of 75 (80%) tweets by government institutes, 124 of 201 (61.7%) tweets by physicians, and 42 of 101 (41.6%) tweets by dieticians were true.

More than half of the health-related tweets from nonofficial health institutes (169/248, 68.1%) and dietician accounts (59/101, 58.4%) were false. Tweets by the physicians were more likely to be rated as “true” compared to other groups (*P*<.001) (Table 3).

**Table 3:** Comparative analysis of account type and final validity of tweets.

Type of account	Final opinion, n (%)^a^	
	False n=320	True n=305
Government institute	15 (20.0)	60 (80.0)
Physician	77 (38.3)	124 (61.7)
Nonofficial health institute	169 (68.1)	79 (31.9)
Dietician	59 (58.4)	42 (41.6)

^a^ For 4×2 table, *P*<.001.

## Discussion

A comparative analysis of the 625 health-related Arabic-language tweets showed that 60 of 75 (80%) tweets by government institutes, 124 of 201 (61.7%) tweets by physicians, and 42 of 101 (41.6%) tweets by dieticians were evidence based. More than half of the health-related tweets (169/248, 68.1%) from nonofficial health institutes were false and tweets by the physicians were more likely to be rated as true compared to other groups (*P*<.001).

Twitter is an online minefield of health-related information, which can considerably affect patient health. It allows for seamless patient-physician relationships and access to infinite online discussions and information on health-related topics.

With the advent of social media, health advice and recommendations from the Internet can influence a patient apart from their physician. Patients are becoming increasingly aware of treatment options, health literacy, and knowledge about disease. Health-related information online is especially beneficial for patients who are immobile and homebound as a result of debilitating illness [7]. However, the Web can also be used to foster unscientific health messages; therefore, patients who are unable to distinguish valid tweets from invalid ones may be misguided. Thus, health care professionals need to be vigilant and responsible for health-related information posted on the Internet [8].

Furthermore, a group needs to be created including government institutes, physicians, other health care professionals, and researchers to ensure that online health care resources are current, credible, and reliable for patient use. This information should be available in a format that is user-friendly, comprehensible, and easily accessible. The use of valid evidence-based Web resources can ensure patient-friendly formats [8,9].

This is the first study to review health-related tweets posted in Arabic language and is comparable to other reviews of health-related reviews in English [10]. The results of this study show that the content of tweets by health-related users on Twitter varied with user type (ie, the government institutes share most of evidence-based medical tweets). Also, in this study 1 in 3 physicians shared health information that was rated false in contrast with previous studies that showed physicians shared testable claims.

This study also shows the reviewers achieved moderate levels of agreement (0.78-0.22) in the classification of tweets as true or false. This could be attributed to expert 2, whose “true” votes were higher than the other 2 experts. This highlights the need for further discussions between the experts regarding classifying the health-related tweets.

### Limitations

The selection of the sample size was limited by health-related tweets in Arabic language on Twitter; thus, it was not feasible to select a random sample of all health users and their tweets reducing the generalizability of the study results.

This study included categorization of health-related tweets by user account followed by analysis. However, the user category designated as “nonofficial health institutes” consisted of accounts whose background was not verifiable and may not be from health-related users. Thus, the results likely underestimate potential differences between groups, emphasizing the need for in-depth analysis.

In addition, user accounts were neither verified independently nor checked against other databases. Furthermore, the low interobserver agreement is attributed to each reviewer’s perception of evidence based, rather than based on evidence, which limits the validity of the results.

This study gives two clear and simple messages to health care professionals, patients, and the general public who access Arabic medical tweets. Firstly, the medical information obtained from accounts on Twitter needs to be confirmed with evidence before applying to real-life situations. Secondly, the scientific value of the tweets from government institutes and physicians is higher compared with other users.

### Future Recommendations

The findings of this study set a baseline for future analyses. Our study recommends developing a consensus around the types of tweets physicians should send based on a minimal evidence level that should be included in the tweet. In addition, health care professionals need to work toward creation of guidelines and policies on the use of social media in modern health care.

### Conclusions

Approximately half of the medical tweets from professional accounts on Twitter were found to be false based on expert review. Furthermore, most of the evidence-based health-related tweets were posted by government institutes and physicians.

## Abbreviations

RCT: randomized controlled trial

## Multimedia Appendix 1

Arabic search terms.
