# Supplementary material for: Are Health-Related Tweets Evidence Based? Review and Analysis of Health-Related Tweets on Twitter
Source: J Med Internet Res. 2015 Oct 29;17(10):e246. doi: 10.2196/jmir.4898 (PMC4642373; doi:10.2196/jmir.4898)
Supplement: Supplementary file 1 [file jmir_v17i10e246_app1.pdf]

Methods

Identification of relevant Twitter accounts

The relevant accounts were identified via a 4-step process. The first step involved a search of the Twitter website (www.twitter.com) using the following search terms in Arabic: health (صحة), your health (صحتك), agility (رشاقة), regimen (رجيم), healthy diet (صحي اكل), drugs (ادوية), disease (مرض), diseases (امراض), drug (دواء), treatment (علاج), prohibited drugs (طبية محظورات), epidemic (وباء), inflammations (التهابات), infection (عدوى), medical information (معلومات طبية), doctors (اطباء), hospitals (مستشفيات), daily medical information (يومية طبية معلومة), nutrition (تغذية), medical accounts (طبية حسابات), health accounts (صحية حسابات), doctor accounts (اطباء حسابات), and nutrition accounts (تغذية حسابات).
